# Exposing the Specific Roles of the Invariant Chain Isoforms in Shaping the MHC Class II Peptidome

**DOI:** 10.3389/fimmu.2013.00443

**Published:** 2013-12-13

**Authors:** Jean-Simon Fortin, Maryse Cloutier, Jacques Thibodeau

**Affiliations:** ^1^Laboratoire d’Immunologie Moléculaire, Département de Microbiologie, Infectiologie et Immunologie, Université de Montréal, Montréal, QC, Canada

**Keywords:** invariant chain, p35, di-leucine motif, di-arginine motif, MHCII trafficking, antigen presentation, MHCII

## Abstract

The peptide repertoire (peptidome) associated with MHC class II molecules (MHCIIs) is influenced by the polymorphic nature of the peptide binding groove but also by cell-intrinsic factors. The invariant chain (Ii) chaperones MHCIIs, affecting their folding and trafficking. Recent discoveries relating to Ii functions have provided insights as to how it edits the MHCII peptidome. In humans, the Ii gene encodes four different isoforms for which structure-function analyses have highlighted common properties but also some non-redundant roles. Another layer of complexity arises from the fact that Ii heterotrimerizes, a characteristic that has the potential to affect the maturation of associated MHCIIs in many different ways, depending on the isoform combinations. Here, we emphasize the peptide editing properties of Ii and discuss the impact of the various isoforms on the MHCII peptidome.

The invariant chain (Ii; CD74) has multiple functions but is best characterized as the main MHC class II (MHCII) chaperone. Ii is a type II protein consisting of a short cytoplasmic tail, a transmembrane region and a luminal domain that can be further partitioned into a membrane-proximal disordered region, the main MHCII-interacting sequence (CLIP), and a C-terminal trimerization domain (1, 2). Mice express two Ii isoforms, p31 and p41, the latter resulting from alternative splicing (3). In humans, the corresponding isoforms are known as p33 and p41. Additionally, around 20% of the Ii mRNAs are translated from an upstream start codon that generates the p35 and p43 isoforms. These bear a 16-amino acid cytoplasmic extension including a strong di-arginine (RxR) ER retention motif (4–6).

Synthesized alongside MHCIIs, Ii can be viewed as: (i) a GUARDIAN that controls access to the MHCII groove; (ii) a SCAFFOLD that assists folding and pairing of α and β MHCII chains; and (iii) a LEADER that directs MHCIIs to the endosomal pathway. It is well established that these Ii functions depend primarily on the ability of its CLIP region to occupy the peptide groove of MHCIIs. Numerous reports showed that Ii proteolysis in endosomes allows HLA-DM to free the groove of CLIP and to catalyze the binding of nominal antigenic peptides [reviewed in Ref. (7)]. Herein, we describe the main chaperone functions of Ii and discuss how the various isoform-specific features can modulate its peptidome-editing properties (Figures 1A–D).

**Figure 1:**
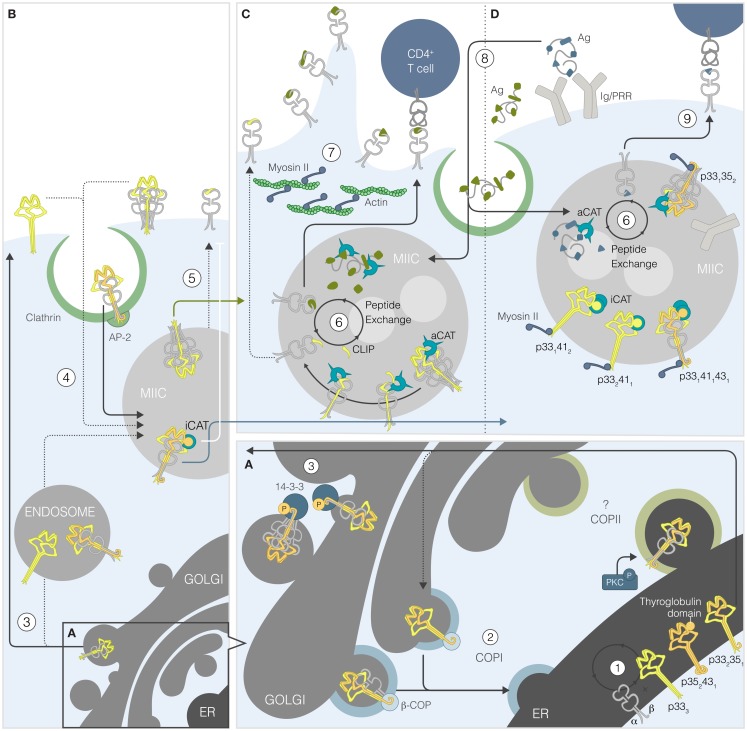
**Portrait of the role of the human invariant chain in MHCII presentation**. **(A)** MHCII α and β chains assemble with Ii in the ER [1]. The four Ii isoforms randomly associate into trimers, some of which bear ER retention motif(s) and/or thyroglobulin domain(s). Unphosphorylated Iip35/p43-containing trimers, associated with MHCIIs or not, exit the ER but are recognized by β-COP and undergo retrograde transport [2]. The MHCII-bound Ii that gets phosphorylated by PKC binds 14-3-3β, thereby preventing β-COP binding and allowing anterograde transport [3]. **(B)** From the Golgi, the different complexes gain access to the plasma membrane or early endosomes [3]. The complexes at the plasma membrane reach the MHCII-rich compartment (MIIC) after being internalized into clathrin-coated pits [4]. In endosomes, presence of p41/43 will reduce processing by inhibiting cathepsin S (iCAT) and slowing-down Ii processing and/or transport to the cell surface [5]. **(C)** In multivesicular MIICs, the carboxy-terminal trimerization domain of Ii is cleaved by non-cysteine proteases, generating the p22 fragment. Then, cysteine proteases remove the glycosylated portion to form the p10 fragment before active cathepsin S (aCAT) cuts the anchored portion, leaving CLIP in the MHCII groove. CLIP is then exchanged for an antigenic peptide spontaneously or by DM [6]. Ii degradation frees myosin II, which can restore the cell motility and remodeling of endosomes [7]. **(D)** Antigens are internalized by pinocytosis or receptor-mediated endocytosis and degraded by proteases, including cathepsins [8]. In the presence of p41/43, processing is more focused given the inactivation of cathepsins. Thus, the MHCIIs that gain access to the plasma membrane present peptides derived from receptor-mediated Ag internalization to CD4^+^ T cells [9].

## Guardian

Early results on the role of Ii have revealed its importance in the presentation of intact Ags (8). By guiding MHCIIs to endosomes while blocking their Ag-binding groove, Ii allows MHCIIs to gain access to peptides from processed Ags and thus influences the pool of associated-peptides (9). This is best exemplified by the differential reactivity of a panel of autoreactive T cell clones co-cultured with Ii^+^ or Ii^−^ APCs (10). Indeed, the content of the MHCII groove differs in transfected cell lines whether or not Ii is expressed. In a mass spectrometry analysis of DR4-eluted peptides, lack of Ii biased the peptide origin toward cytoplasmic proteins, whereas Ii allowed the binding of peptides derived from exogenous and endocytic proteins (11). Also, the repertoire is strongly skewed in Ii KO mice, as demonstrated by mixed lymphocyte reactions and aberrant CD4^+^ T cell selection (12–18). In humans however, little is known on the impact of Ii deficiency in different cell types. The effect of Ii on the peptidome varies and following its degradation, the residual CLIP peptide also affects the peptide’s assortment to be presented to T lymphocytes. Indeed, a series of articles from Mellins and collaborators describing the MHCII-CLIP affinity relationship suggest that poor Ii and CLIP chaperoning leads to Ag processing defects with the potential to instigate MHCII-associated autoimmunity (19–23).

In the absence of Ii, both mouse and human MHCIIs bind a collection of long polypeptides, most likely originating from misfolded ER proteins (24–26). Interestingly, even in the presence of Ii, it was recently reported that MHCIIs displayed some ER polypeptides at the plasma membrane, the latter competing with Ii for the class II binding groove (27). Despite the fact that MHCIIs can associate with ER polypeptides, there are numerous functional examples of endogenous and exogenous CD4 T cell epitopes that are presented in the absence of Ii expression [reviewed in Ref. (28)]. While presentation of some of these peptides was negatively affected by the presence of Ii, others were Ii-independent and loaded on recycling MHCIIs (29, 30). Ii is usually produced in vast excess and most if not all MHCIIs mature in association with Ii (31). Still, it is tempting to speculate that under some physiological conditions, the Ii/MHCII protein ratio may decrease sufficiently to impact the peptidome. Accordingly, knocking down Ii in cancers represents a possible therapeutic avenue, allowing these cells to present new antigens to tumor infiltrating T cells (32, 33).

Beside the gross peptidome alterations noted in the non-physiological absence of Ii, subtle variations have been observed when MHCIIs were expressed in the context of specific Ii isoforms. The GUARDIAN role of Ii is not exclusively CLIP-centered but also shaped by the p41/43 thyroglobulin domain, which regulates the proteolytic activity of numerous cathepsins (18, 34–37). Among them, CatS breaks down large polypeptides and provokes MHC/Ii complex dissociation by cleaving Ii between the transmembrane and CLIP regions in APCs (38). In thymic epithelial cells, CatV (CatL in mice) occupies this role although redundancy between cathepsin family members is observed (39, 40). As a result, MHCIIs are freed from Ii cytoplasmic targeting and may egress to the plasma membrane. It has also been suggested that the effect of p41/43 is echoed to surrounding Ii (p33/35), limiting overall processing, and that the thyroglobulin domain chaperones cathepsins, increasing half-life and maintaining a pool of mature enzymes in the MIICs (41, 42). The proportion of p41/43 isoforms varies from 10 to 40% in professional APCs and this provides a mechanism to modulate cathepsin activity (43). Lastly, it has been shown that Ii luminal domain is involved in increasing the half-life of MHCII by delaying endosomal maturation (44). As a result, the pool of antigenic peptide could be skewed toward receptor-mediated protein intake taking place in the highly specialized MIICs (Figure 1D). To efficiently protect the cell from aberrant Ag presentation, the Ii isoforms must work as a team. As seen in Tg mice expressing exclusively one mouse or human Ii isoform, whether p31, p35 or p41, Ag presentation, and CD4 T cell selection can be restored (18, 45–48). Still, Ag presentation by alternatively spliced isoforms was not equivalent, suggesting the existence of divergent pathways (18, 34). Also, the differential outcomes of experimental allergic encephalomyelitis (EAE) or asthma in p31- vs p41-expressing mice point to distinct class II peptide repertoire (45, 49). Along the same lines, it was reported that NOD mice devoid of Ii are protected from developing type 1 diabetes (50). The isoform balance is put into perspective by Baugh et al., which demonstrated that the onset of experimental EAE and rheumatoid arthritis are delayed when cathepsin S inhibitors are administered to mice (51). Further study of the distinct roles of Ii is much needed to understand the implication of the various isoforms in immunity. Altogether, these observations clearly demonstrate the general impact of Ii on the MHCII peptidome and the subtle editing role of the exon 6b-encoded domain.

## Scaffold

While transfected MHCIIs could egress from the ER in cell lines, Ii was found to favor the pairing and trafficking of haplotype-matched and -mismatched α and β chains (52, 53). Some key properties of Ii became apparent with the characterization of Ii-deficient mice, although cell-type- and haplotype-dependent differences were reported (54). A detailed sequence of events leading to formation of the MHCII/Ii complex was first described by Cresswell and collaborators (Figure 1A) (55). According to the model proposed by Roche et al. three assembled MHCII αβ heterodimers associate sequentially with a preformed Ii trimer, generating a pentamer, then a heptamer, and ultimately a nonamer with the ability to egress from the ER (56). This dogma was later refined to include intermediate steps such as the initial binding of Ii to an MHCII α chain prior to the pairing of an isotype-matched β chain (57, 58). Although a variety of chaperones such as BIP and calnexin have been shown to interact with the MHCII Ag presentation machinery, their exact role in the final assembly and ER egress of the MHCII/Ii complex is not well defined (55, 59). Furthermore, the interaction between calnexin and MHCIIs until the final nonamer formation suggested that egress is tightly restricted (60). However, given the existence of transport-competent heptamers and pentamers, it does not appear that universal quality control mechanisms are in place to prevent egress of non-stoichiometric complexes (60–62). As MHCII-free Ii trimers can egress from the ER, the relative abundance of MHCIIs and Ii likely influences the complex stoichiometry. Cell-type-specific differences and the affinity of CLIP for the MHCII groove may come into play as well.

In humans, although p33 is the most abundant isoform and generates some homotrimers, it is mostly part of heterotrimers that have also incorporated an RxR-containing moiety (63–65). By analogy to other multi-protein complexes such as the K_ATP_ channel (66), the di-arginine motif would prevent premature ER egress of MHCII-unsaturated Ii trimers (i.e., pentamers and heptamers). Indeed, p35/43 require binding of the MHCIIβ chain for anterograde trafficking (67–69). Although less abundant than their respective Iip33/p41 counterparts, p35/43 are dominant as the stochastic incorporation of a single RxR-bearing Ii moiety will prevent ER egress of an heterotrimer (70, 71). Thus, p35/p43 will favor the formation of high-order MHCII/Ii oligomers. Indeed, as p35/43 both need to be phosphorylated by PKC and be associated with MHCIIs to become transport competent, they form larger complexes and egress less efficiently than homotrimers devoid of RxR-containing subunits (Figure 1A) (65, 72, 73). A MHCII molecule binding a p33_2_p35_1_ heterotrimer would have only one chance out of three to egress the ER as a pentamer. This of course is assuming that the MHCII cannot mask the RxR motif in *trans*. This is an important issue as it was recently suggested that steric hindrance caused by the plasma membrane and the bending of the MHCII/Ii complex only allows formation of pentamers (61). If this holds true, we have to assume that a *cis* interaction between the MHCII and p35/43 is not required to overcome the retention motif, otherwise many doomed complexes would be formed.

The advantage, if any, conferred by the presence of an ER retention motif in p35/43 remains obscure. A variable Ii/MHCII stoichiometry may modulate the MHCIIs turnaround and thus, the peptidome that is displayed to T cells. One can imagine that although Ii is in excess, its retention of Ii increases the available ER pool and ensures that the ratio of free over Ii-bound MHCIIs is as low as possible. This way, most MHCIIs would acquire their final cargo in the endocytic pathway instead of the ER. Whether or not the cell can modulate its physiology to favor the binding of endogenous ER peptides remains to be seen.

## Leader

The fundamental functional distinction between MHCI and MHCII molecules comes from the fact that they acquire peptides in different locations (74). The seminal studies of Ziegler and Unanue demonstrated that the presentation of CD4 T cell epitopes by MHCIIs was inhibited by chloroquine, highlighting the importance of low pH compartments (75, 76). Evidence for a role of Ii in the trafficking of MHCIIs to endosomes has been described in numerous reviews (55, 77, 78). In the absence of Ii, MHCIIs are not transported to endocytic compartments as efficiently and accumulate at the plasma membrane (4, 5). Confocal and electron microscopy experiments using transfected cell lines revealed that a clear colocalization of MHCIIs with endosomal markers or internalized antigens required co-expression of Ii (70, 79). Deletion and site-directed mutagenesis experiments established the importance of the cytoplasmic domain for intracellular trafficking and allowed the mapping of two classical leucine-based endosomal sorting signals in all Ii isoforms (79).

In line with the role of the leucine-based motifs in Ii degradation and CLIP removal, it was shown that deletion of the Ii cytoplasmic tail resulted in the cell-surface display of Ii/MHCII complexes being unable to acquire antigenic peptides (80). In contrast, one can wonder if the specific characteristics of p35/43 affect transport of the complex and, ultimately, the peptidome. Many studies using various Ii^+^ cell types and transfected cell lines have reported that even in the absence of MHCIIs, some p33/p41 homotrimers gain access to post-Golgi compartments and acquire complex N- and O-linked oligosaccharides (4, 5, 68, 81). However, as mentioned above, p35/p43-containing trimers are retained in the ER (63). The general model stipulates that an unphosphorylated p35 moiety binds β-COP upon arrival at the cis-Golgi sorting station, causing the retrograde transport of the complex in COPI-coated vesicles and the apparent steady-state ER retention (82) (Figure 1A). However, when phosphorylated by PKC on serine 8, Ii recruits 14-3-3β to prevent the binding of β-COP on the RxR motif (47, 56–58, 73). Still, it remains to be determined how the complex is transported from the ER to the Golgi. While largely undefined, export signals have been described in some cargo proteins, allowing their incorporation in COPII-coated transport vesicles originating at ER exit sites (83). Other transmembrane proteins exit through the default pathway (84). Whether the 16-amino acid extension of p35/43 confers specific sorting properties to MHCIIs in such early step as ER egress has yet to be addressed. Another important question that remains is, if the RxR motif is masked by 14-3-3β, why can’t a phosphorylated Ii trimer be released from the Golgi in MHCII-negative cells? Although there is compelling evidence for competition between 14-3-3β and β-COP, the need for MHCIIs in the transport of p35/p43-containing complexes beyond the Golgi apparatus was overlooked in previous studies and remains unexplained.

The stringent quality control mechanism operating at the level of the Golgi suggests that p35/p43-including complexes do not simply reach the plasma membrane through the default pathway. Many groups have studied the route taken by the MHCII/Ii complex to reach the late endosomes/lysosomes [reviewed in Ref. (85)] (Figure 1B). It is now recognized that AP-2 adaptors, which connect cargo and plasma membrane clathrin-coated pits, are important in the sorting of MHCII/Ii complexes to the endocytic pathway. The actual model proposes that the bulk of MHCII/Ii complexes exit the Golgi by a clathrin-independent mechanism en route to the plasma membrane where they are internalized in association with AP-2 [Ref. (86, 87) and references therein). However, one must bear in mind that there could be important cell-type differences in the transport of MHCIIs. Also, in some of the studies looking at the trafficking of Ii, it is not entirely clear which Ii isoform(s) was (were) expressed and in what proportions. Thus, a thorough comparison of p35 and p33 trafficking is much needed.

One clear difference in the trafficking of p33 and p35 is that the latter is not detected at the plasma membrane (71, 72). Kuwana et al. have shown in transfected cells that a dominant-negative form of dynamin caused the cell-surface display of p35, suggesting that in fact, p33 and p35 follow the same path to endosomes (72). The reason why the internalization kinetics of phosphorylated p35 is increased as compared to p33 is not known but may relate to its affinity for AP-2 (88).

Many groups have documented the impact of Ii on the endocytic pathway [see Ref. (78)] (Figure 1C). Ii-expressing cells accumulate large endosomes, in which Ag and MHCIIs degradation is slowed (89–93). Such effects are dependent on the cytoplasmic tail and the luminal trimerization domain common to all Ii isoforms. However, based on studies using cathepsin KO mice, it became clear that the Ii thyroglobulin domain exerts further pressure on MHCII trafficking and maturation by limiting cathepsin-mediated degradation while preventing cathepsin-sensitive epitopes from proteolysis (94–96). Surprisingly, p41/43 and cathepsins were shown to colocalize in compartments not implicated in Ag presentation, a finding suggesting a role in phagocytosis rather than Ag processing (97). In light of the recent results by Faure-André et al. describing Ii-myosin II interactions, it seems that Ii is also involved in cell motility/remodeling (98) (Figures 1C,D). Reduced Ii processing caused by p41/43 would increase the MIIC’s interaction with the myosin II motor, providing necessary extraction force to internalize membrane Ag in these compartments (99). The endocytic pathway is a complex system made of different tubular/vesicular entities and the exact location where MHCIIs acquire peptides is still debated (100, 101). It is unknown if p35/p43 have additional modulatory properties that could translate into a change in the peptidome and the contribution of each isoform to the endocytic landscape covered by Ii remains to be evaluated (102, 103).

## Concluding Remarks

The list of functions ascribed to Ii is continuously growing. Beside its many roles in MHCII Ag presentation, Ii was shown to chaperone other presentation molecules, such as CD1d. As this class 1b molecule acquires its ligands in the endocytic pathway, the role of Ii in the selection of lipid Ags is of great interest (104). Recently, Ii was shown to have a key impact in cross-presentation, suggesting that its isoforms may fine tune the peptide repertoire associated with MHCI molecules in DCs (105).

The Ii pool is highly heterogeneous and an important question that remains is the potential isoform-specific influence of post-translational modifications such as the addition of a glycosaminoglycan (chondroitin sulfate, CS) side chain in the ER/Golgi (106). Ii-CS binds CD44 and can enhance T cell responses (107). Given that p35/p43 regulate surface display of Ii trimers (71), it would be interesting to determine in humans the contribution of these isoforms on the chaperone-independent functions of Ii, such as being a cell-surface receptor for the macrophage migration inhibition factor (MIF) and *Helicobacter pylori* (108). Whether alternative splicing affects the affinity of these ligands for the Ii receptor remains to be measured. Interestingly, the cytoplasmic tail is pivotal in the capacity of Ii to transduce signals in response to MIF or after endosomal cleavage of its transmembrane region by Sppl2a (109–111). The potency of p35/p43 in this context should be tested.

The existence of Ii isoforms offers multiple layers of control over Ag presentation. Major transformations occur during the activation of APCs following, for example, microbial activation of pattern recognition receptors (PRRs) signaling pathways. If mediators of the inflammatory response (IFN-γ, TNF-α, IL-10 etc) can modulate the behavior of Ii isoforms and if the ensuing changes in the expression levels of MHCIIs or Ii can affect the peptidome should be systematically addressed. Evidence that the relative proportions of these isoforms can somehow be regulated comes from the study of chronic lymphocytic leukemia in which overexpression of p35 has been reported (112, 113). Whether p35 plays a role in tumor escape from the immune system by modulating the peptidome remains to be determined. Also, differential p35 expression between B cells from monozygotic twins discordant for type 1 diabetes was shown to affect Ag presentation and could potentially contribute to the development of the disease (114). On a final note, expression of Ii-Ag fusion proteins in APCs represents a potential immunization strategy that targets Ags directly to endosomes and skews the peptidome (115, 116). Alternatively, recombinant proteins have been engineered by replacing CLIP with the sequence of a T cell epitope (117). The efficacy of these promising vaccine approaches may benefit from the study of the biology of the various Ii isoforms.

## Conflict of Interest Statement

The authors declare that the research was conducted in the absence of any commercial or financial relationships that could be construed as a potential conflict of interest.
